# Long non-coding RNA SNHG5 regulates ulcerative colitis via microRNA-375 / Janus kinase-2 axis

**DOI:** 10.1080/21655979.2021.1953219

**Published:** 2021-08-02

**Authors:** Hui Li, Ji Xuan, Wei Zhang, Zhentao An, Xinyu Fan, Min Lu, Yaozhou Tian

**Affiliations:** a Department of Gastroenterology, Affiliated Hospital of Integrated Traditional Chinese and Western Medicine, Nanjing University of Chinese Medicine, Nanjing, Jiangsu, China; b Department of Gastroenterology, Jinling Hospital, Nanjing, China; c Department of Preventive Treatment, Affiliated Hospital of Integrated Traditional Chinese and Western Medicine, Nanjing University of Chinese Medicine, Nanjing, Jiangsu, China

**Keywords:** Ulcerative colitis, apoptosis, lncRNA snhg5, miR-375, *jak2*, tnf-α

## Abstract

Ulcerative colitis (UC) is an intestinal inflammatory disorder. Long non-coding RNAs (lncRNAs) are collectively involved in UC. This study is designed to explore the roles of lncRNA (small nucleolar RNA host gene 5) SNHG5 in UC. Gene or microRNA (miRNA) expression was detected using RT-qPCR and western blot, respectively. Cellular functions were analyzed by cell counting kit 8 (CCK8), 5-ethynyl-2′-deoxyuridine (EdU) assay, flow cytometry, and the terminal deoxyribonucleotidyl transferase (TDT)‐mediated dUTP‐digoxigenin nick end labeling (TUNEL) assays. Lactate dehydrogenase (LDH) content was determined by a cell cytotoxicity assay. The interactions between miR-375 and SNHG5 or Janus kinase-2 (*JAK2*) were verified by a luciferase reporter assay. SNHG5 was up-regulated in intestinal mucosa tissues of UC patients as well as tumor necrosis factor alpha-treated (TNF-α-treated) young adult mouse colon (YAMC) cells. Down-regulated SNHG5 promoted cell proliferation and inhibited apoptosis of YAMC cells. miR-375 was verified to be a target of SNHG5 and was suppressed by TNF-α treatment in YAMC cells. Over-expression of miR-375 restored YAMC cellular functions. Additionally, miR-375 targeted *JAK2*, which was up-regulated by TNF-α treated YAMC cells. Up-regulation of *JAK2* induced the dysfunction of YAMC cells. Knockdown of SNHG5 promoted the proliferation and suppressed the apoptosis of YAMC cells via regulating miR-375/*JAK2* axis. Therefore, knockdown of SNHG5 may be a promising therapy for UC.

## Graphical Abstract

**Figure:**
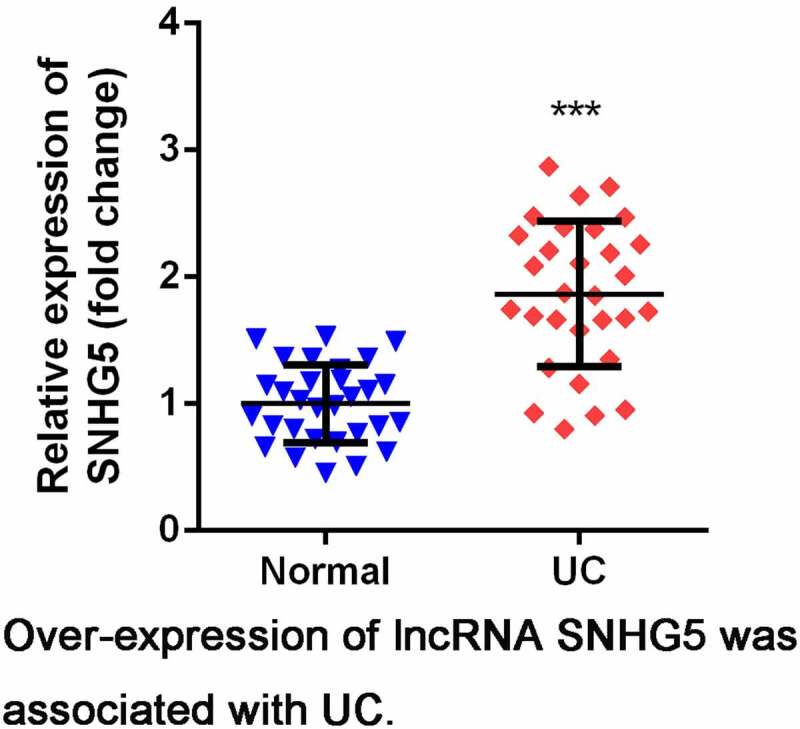

## Introduction

Ulcerative colitis (UC) is an intestinal inflammatory disorder, which occurs most often in adults in their 30s [1,2]. Varieties of drugs for individualized treatment plus regular surveillance colonoscopies are the recognized regular therapeutic treatments for UC patients [3]. Various factors complicated the pathogenesis of UC. Inflammatory response in intestinal epithelial cells promotes the pathogenesis of UC [4,5]. Tumor necrosis factor alpha (TNF-α) collectively participates in inflammatory bowel disease [6]. What’s more, anti-TNF-α drugs have been widely used to treat UC in clinic to minimize steroid exposure and dependence in recent years [7,8]. Surgical rates for UC, however, are not decreased under the application of anti-TNF-α drugs [7]. Thus, it is urgent to study the potential molecular mechanism of TNF-α on UC.

Long non-coding RNAs (lncRNAs), without protein-encoding capability, function as the competing endogenous RNAs (ceRNAs) by sponging microRNAs (miRNAs) to regulate the progression of various diseases [9,10]. Recently, numerous lncRNAs are associated with the onset and development of UC, such as lncRNA MALAT1 [11], lncRNA CDKN2B-AS1 [12], and lncRNA TUG1 [13]. lncRNAs may have the potential as biomarkers and therapeutic targets. lncRNA SNHG5, located on chromosome 6q14.3, was reported to regulate progression of colorectal cancer [14–16], nasopharyngeal carcinoma [17], ovarian cancer [18], breast cancer [19], glioma [20], and COPD [21], etc. What’s more, SNHG5 was down-regulated in colitis samples according to bioinformatic analysis [22]. Despite of the above literature, the mechanisms of SNHG5 have not previously been elucidated in UC.

miRNAs are considered to take part in various diseases via targeting the downstream genes [23]. Dysregulated miR-375 contributes to the progression of oral squamous cell carcinoma [24], prostate cancer [25], CRC [26], and osteosarcoma [27]. Furthermore, miR-375 was down-regulated in UC [28,29]. In this study, the potential roles of SNHG5 and miR-375 in UC were investigated.

Our study aimed to investigate the expression as well as molecular mechanism of SNHG5 in the development of UC. Furthermore, we hypothesized that SNHG5 sponges miR-375 to regulate *JAK2* expression to regulate proliferation and apoptosis of UC cell. Our research provided a novel understanding of UC treatment.

## Materials and methods

### Clinical samples

A total of 30 patients with UC diagnosed at Affiliated Hospital of Integrated Traditional Chinese and Western Medicine, Nanjing University of Chinese Medicine, from January 1, 2019, to May 1, 2020, were obtained. Thirty healthy volunteers who underwent colonoscopy in the same period were selected as controls. Intestinal mucosa tissues were taken from sigmoid colons. Clinical samples were rapidly stored at in liquid nitrogen at −80°C. This study was authorized by the Ethics Committee of Affiliated Hospital of Integrated Traditional Chinese and Western Medicine, Nanjing University of Chinese Medicine. All the patients had provided the informed consent.

### Cell culture and transfection

The conditionally immortalized epithelial cell line of young adult mouse colon (YAMC) cell was purchased from Institute of Hematology, Chinese Academy of Medical Sciences (Tianjin, China) and cultured in complete Dulbecco’s modified Eagle medium (DMEM) supplemented with 10% fetal bovine serum (FBS) (Gibco, Calif, USA) and 1% penicillin/streptomycin (Thermo Fisher, Calif, USA). YAMC cells were exposed to 10 ng/ml TNF-α (10 μG; H8916-10UG; Sigma-Aldrich, NJ, USA) for 12 h to establish a model of UC in *vitro* [13].

si-SNHG5 1#, si-SNHG5 2#, miR-375 inhibitor, NC inhibitor, miR-375 mimic, *JAK2*, and the negative control or empty vector were transferred into cells using Lipofectamin^TM^ 3000 (Life Technologies, Calif, USA).

### Real-time quantitative reverse transcription-polymerase chain reaction (qRT-PCR)

qRT-PCR was used to evaluate mRNA and miRNA levels [30]. Total RNA of YAMC cells extracted by TRIzol® reagent (Thermo Fisher Scientific, Calif, USA). Reverse transcription and qPCR were carried out using BlazeTaq One-Step SYBR Green RT-qPCR Kit (with ROX) (QP071; GeneCopoeia, MD, USA) on SEDI Thermo Cycler controlled by the Control Bus Net software package (Wealtec Bioscience, Taiwan, China). *GAPDH* and *U6* served as internal reference for mRNA and miRNA, respectively. The results were calculated using 2^−ΔΔCt^ method. The primers used in PCR were obtained from Nanjing Genscript Biotech Co., Ltd (Jiangsu, China). The primer sequences were listed in Table 1.

**Table 1.** The sequences for primers used in this study

Gene	Sequence	
SNHG5	Forward	5ʹ-TACTGGCTGCGCACTTCG-3’
	Reverse	5ʹ-TACCCTGCACAAACCCGAAA-3’
miR-375	Forward	5ʹ-GTGCAGGGTCCGAGGT-3’
	Reverse	5ʹ-AGCCGTTTGTTCGTTCGGCT-3’
*GAPDH*	Forward	5ʹ-AGGTGAAGGTCGGAGTCAACG −3’
	Reverse	5ʹ-AGGGGTCATTGATGGCAACA-3’
*JAK2*	Forward	5ʹ-TCTGGGGAGTATGTTGCAGAA-3’
	Reverse	5ʹ-AGACATGGTTGGGTGGATACC-3’
*U6*	Forward	5ʹ-CTCGCTTCGGCAGCACATATACT-3’
	Reverse	5ʹ-ACGCTTCACGAATTTGCGTGTC-3’

### Cell counting kit 8 (CCK8)

Cell viability was determined by a CCK8 assay [31]. YAMC cells were plated in a 96-well plate (1 × 10^4^ cells/well). 10 μl of CCK8 reagents (AMJ-KT0001; AmyJet Technology, Beijing, China) were added to each well of the plate. Then, cells were cultured in the incubator. The absorbance values were evaluated with a microplate reader (HBS-1096 C; Nanjing DeTie Experimental Equipment, Jiangsu, China) at the wavelength of 450 nm.

### 5-ethynyl-2′-deoxyuridine (EdU) assay

The EdU assay was performed to determine cell proliferation [32]. 2 × 10^5^ YAMC cells were seeded into a 24-well plate with 200 μL of diluted EdU (Beyotime, Jiangsu, China). Then, cells were washed with PBS twice, 200 μL of Hoechst was applied, and were cultured in the dark for 30 min. Finally, EdU-labeled and Hoechst stained cells were captured.

### Cell cytotoxicity assay

The levels of lactate dehydrogenase (LDH) in YAMC cell were detected with a LDH Assay Kit (Abcam, Calif, USA) to evaluate cell cytotoxicity [33].

### Flow cytometry assay

The apoptosis of logarithmic phase YAMC cells was detected by a Annexin V-FITC/PI kit (Yeason Biotech, Shanghai, China) [34]. The supernatant was discarded after YAMC cells were centrifuged. Cells were stained with Annexin V-FITC and PI. Finally, the apoptosis rates were detected by a flow cytometry.

### Terminal deoxyribonucleotidyl transferase (TDT)‐mediated dUTP‐digoxigenin nick end-labeling (TUNEL) assay

Cell death of YAMC was determined using a TUNEL kit (KGA702-1, KeyGEN BioTECH, Jiangsu, China) [35]. Briefly, cells were deparaffinized and then cultured with TUNEL. Then, cells were counterstained with DAPI. Images were acquired on a Nikon microscope (90i, Tokyo, Japan).

### Western blotting assay

Apoptosis-related proteins including apoptosis inhibitor 5 (API5), caspase-3, and BAX were collected from YAMC cells to evaluate cell apoptosis [36]. Protein concentrations were determined with the BCA kit (Sigma-Aldrich, NJ, USA). Additionally, proteins were isolated by 12% SDS-PAGE gel. The isolated protein was moved onto PVDF membranes (Bio-rad, Calif, USA), which were blocked with 5% nonfat milk. Then, the membranes were incubated with primary antibodies: anti-API5 (70 R-32,287, 1:3000, Fitzgerald, Birmingham, UK), anti-caspase-3 (ab32351, 1:5000, Abcam, Calif, USA), anti-BAX (ab32503, 1:1000, Abcam, Calif, USA), and anti-GAPDH (ab8245, 1:500, Abcam, Calif, USA); and then with secondary antibodies. Finally, protein expressions were captured using an ECL kit.

### Luciferase reporter assay

The interactions between miR-375 and SNHG5 or *JAK2* were verified by a luciferase reporter assay [37]. Full-length 3ʹUTR of SNHG5 and *JAK2* inserted into pMIR-GLOTM luciferase vectors. Afterward, cells were co-transfected with luciferase vectors and empty vector and miR-375 mimic or nc mimic using Lipofectamine® 3000 (Life Technologies, Calif, USA). After 48 h, the results were detected using a Dual-Luciferase Reporter Assay kit (K801-200; BioVision Tech, San Francisco, USA).

### Statistical analysis

Graphpad 6.0 (GraphPad Software, Calif, USA) was used for statistical analysis. Data were presented as ± SD. Student’s t-test (two groups) and one-way ANOVO (multiple groups) were applied for difference analysis. *P < 0.05* was considered statistically significant.

## Results

### Over-expressed SNHG5 was up-regulated in UC

**Figure 1.**
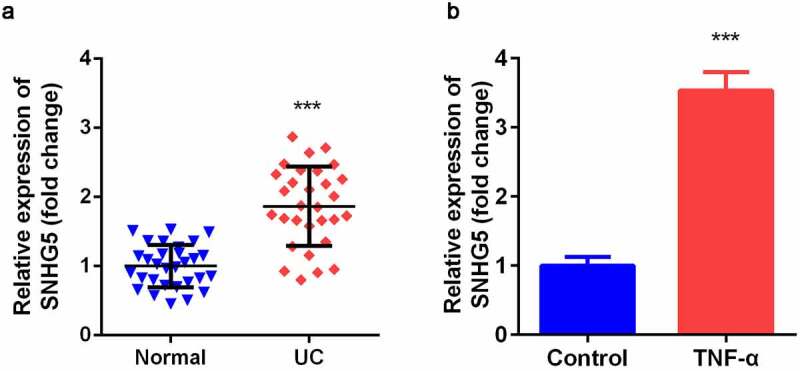
Expression levels of SNHG5 in UC tissues and cells. (a) The expression of SNHG5 in intestinal mucosa tissues. (b) The expression of SNHG5 in YAMC cells. ***p < 0.05

SNHG5 expression level was significantly increased in intestinal mucosa tissues of UC patients compared with normal group (Figure 1a). As shown in Figure 1b, TNF-α-treated YAMC cells significantly up-regulated expression of SNHG5 compared control group.

### Knockdown of SNHG5 modulated cellular functions of YAMC cells

**Figure 2.**
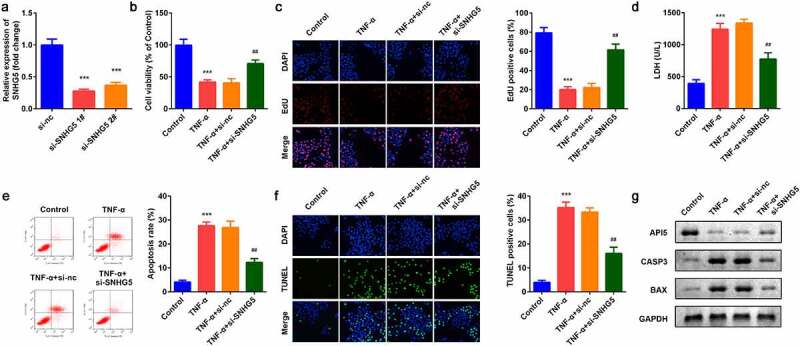
Effects of SNHG5 on YAMC cells. (a) SNHG5 expression levels were detected. (b) Cell viability was detected by CCK8. (c) EdU staining assay was used to determine cell proliferation. (d) Cell injury was examined with the LDH assay. (e) Flow cytometry assay was used to detect apoptosis. (f) TUNEL staining was used to detect cell death. (g) The protein expression of API5, CASP3, and BAX. ***p < 0.001, vs. (negative control) NC group or control group. ##*p* < 0.01, vs. TNF-α group

As shown in Figure 2a, the expression of SNHG5 was decreased after si-SNHG5 plasmids were transferred into YAMC cells, which was more remarkable in si-SNHG5 1# group. TNF-α suppressed cell viability (Figure 2b) and proliferation (Figure 2c) while inhibition of SNHG5 reversed the effects of TNF-α on YAMC cells. Simultaneously, the increase of LDH release induced by TNF-α was significantly suppressed by SHNG5 knockdown (Figure 2d). Furthermore, TNF-α significantly promoted apoptosis while si-SNHG5 neutralized the effects of TNF-α on YAMC cells (Figure 2e-f). SNHG5 knockdown increased the protein expression of API5 and decreased CASP3 and BAX (Figure 2g).

### SNHG5 targeted miR-375 in YAMC cells

**Figure 3.**
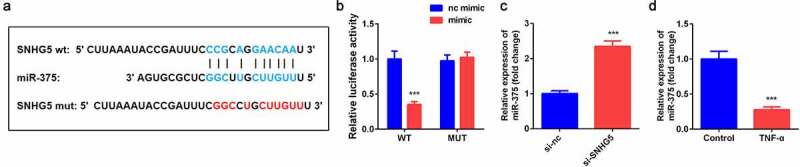
SNHG5 sponged miR-375 in YAMC. (a) Bioinformatics predicted the binding sites between miR-375 and SNHG5. (b) Dual-luciferase reporter assay confirmed that miR-375 was a target of SNHG5 in YAMC. (c-d) The expression of miR-375 was determined by qRT-PCR. ***p < 0.001

The online database Starbase 3.0 predicted that miR-375 was a target of SNHG5 (Figure 3a). Luciferase assay further verified the interaction between miR-375 and SNHG5 (Figure 3b). Furthermore, miR-375 expression was remarkably overexpressed by si-SNHG5 in YAMC cells. Moreover, TNF-α exposure notably decreased the expression of miR-375 (Figure 3c-d).

### Down-regulation of miR-375 reversed the effects of SNHG5 on cell proliferation and apoptosis

**Figure 4.**
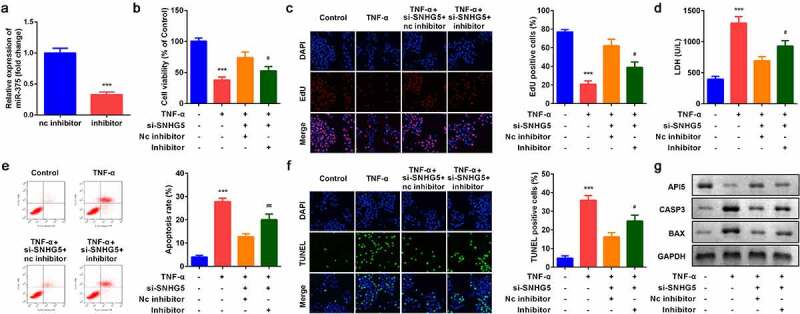
Effects of miR-375 on YAMC cells. (a) miR-375 expression levels were detected. (b) Cell viability was detected by CCK8. (c) EdU staining assay was used to determine cell proliferation. (d) Cell injury was examined with the LDH assay. (e) Flow cytometry assay was used to detect apoptosis. (f) TUNEL staining was used to detect cell death. (g) The protein expression of API5, CASP3, and BAX. ***p < 0.001, vs. NC group or control group. #*p* < 0.05, ##*p* < 0.01, vs. TNF-α group

The expression of miR-375 was remarkably downregulated by miR-375 inhibitor group, suggesting cells were successfully transfected (Figure 4a). Decreased miR-375 notably suppressed cell viability (Figure 4b) and cell proliferation (Figure 4c) and enhanced cell apoptosis (Figure 4e-f). Moreover, Down-regulation of miR-375 reversed the regulatory roles of si-SNHG5 in the release of LDH (Figure 4d). Besides, miR-375 alleviated the effects of SNHG5 knockdown on the expression of API5, CASP3, and BAX in protein level (Figure 4g).

### *miR-375 directly targeted* JAK2

**Figure 5.**
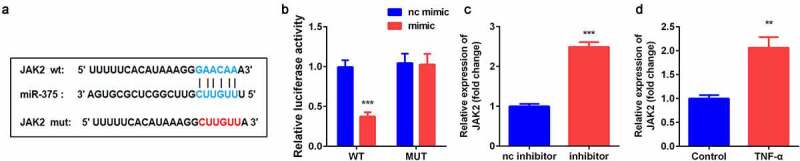
*JAK2* was target gene of miR-375. (a) Bioinformatics predicted the binding sites between miR-375 and *JAK2*.(b) Dual-luciferase reporter assay was conducted to confirm the association between *JAK2* and miR-375. (c-d) The expression of *JAK2* was determined by qRT-PCR. **p < 0.01, ***p < 0.001

TargetScan 7.2 predicted the binding sites between *JAK2* and miR-375 (Figure 5a). Luciferase assay further confirmed the interaction between *JAK2* and miR-375 (Figure 5b). The mRNA expression of *JAK2* was significantly increased by miR-375 inhibitor in YAMC cells. Additionally, *JAK2* was remarkably up-regulated in TNF-α-treated YAMC cells (Figure 5c-d).

### *Up-regulation of* JAK2 *inhibited the effects of miR-375*

**Figure 6.**
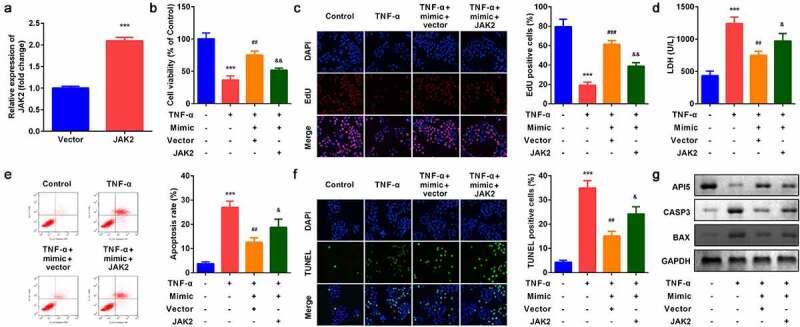
Effects of *JAK2* on YAMC cells. (a) *JAK2* expression levels were detected. (b) Cell viability was detected by CCK8. (c) EdU staining assay was used to determine cell proliferation. (d) Cell injury was examined with the LDH assay. (e) Flow cytometry assay was used to detect apoptosis. (f) TUNEL staining was used to detect cell death. (g) The protein expression of API5, CASP3, and BAX. ***p < 0.001, vs. control group. ##*p* < 0.01, ###*p* < 0.001, vs. TNF-α group. &*p* < 0.05, &&*p* < 0.01, vs. TNF-α + miR-375 mimic group

As shown in Figure 6a, *JAK2* expression was significantly increased after *JAK2* overexpression plasmids were transfeted into YAMC cells. Compared with miR-375 mimic group, *JAK2* apparently inhibited cell viability (Figure 6b) and cell proliferation (Figure 6c) and accelerated apoptosis (Figure 6e-f). Moreover, up-regulation of *JAK2* reversed the regulatory roles of miR-375 mimic in the release of LDH (Figure 6d). Besides, *JAK2* alleviated the effects of miR-375 mimic on the expression of API5, CASP3, and BAX in protein level (Figure 6g).

## Discussion

This study identified the potential roles of lncRNA SNHG5 in UC. We unveiled the underlying mechanisms that SNHG5 promoted the progression of UC via regulating miR-375/*JAK2* axis. SNHG5 was overexpressed in UC. However, knockdown of SNHG5 restored human intestinal epithelial cellular function, the dysfunction of which is one of the key factors for UC. Additionally, SNHG5 sponged miR-375 to promote the upregulation of *JAK2*, which is the key regulator of cytokine and growth factor signaling. Together, SNHG5 overexpression was associated with the development of UC. Knockdown of SNHG5 inhibited the progression of UC via modulating miR-375/*JAK2* axis.

Augmenting evidence reveal that non-coding RNAs are involved in the progression of UC. Long non-coding RNAs are families of RNAs with over 200 nucleotides. Abnormal expression of lncRNAs may function as a promoter or suppressor of UC [11–13]. Dysregulation of SNHG5 plays a crucial role in intestinal diseases. SNHG5 is reported to function as an oncogene in CRC [14]. Moreover, Wang K et al. revealed that SNHG5 was down-regulated in colitis *in vivo* [22]. In this study, SNHG5 was dramatically up-regulated in intestinal mucosa tissues of UC patients and in TNF-α-treated YAMC cells, indicating that SNHG5 may be closely associated with the development of UC. Further study showed that knockdown of SNHG5 restored the function of human intestinal epithelial cells, manifested by enhanced cell proliferation and decreased apoptosis rates. Furthermore, lncRNAs function as ceRNA and modulate cellular functions via sponging miRNAs. For instance, lncRNA CDKN2B-AS1 suppressed inflammatory response in UC through binding to miR-16 and miR-195 [12] Overexpression of TUG1 promoted the proliferation and suppressed the apoptosis of mice intestinal epithelial cell [13].

Therefore, we further identified the target miRNAs of SNHG5. miR-375 was predicted and proved to be a target miRNA of SNHG5. miR-375 was evidenced to be down-regulated in UC [28,29]. It is down-regulation activated inflammatory signaling in UC [28]. Therefore, miR-375 may play a protective role in UC. In this study, miR-375 was decreased in UC. Decreased miR-375 antagonized the effects of SNHG5 and induced dysfunction of human intestinal epithelial cells. These results suggested that SNHG5 may participate in the progression of UC via sponging miR-375.

*JAK2* is an ubiquitously expressed protein involved in the inflammatory response, and is critical in the pathogenesis and progression of diseases [38,39]. TNF-α induced apoptosis was suppressed by inhibiting *JAK2* in osteoblasts [40]. JAK2-STAT3 inhibitor effectively inhibited TNF-α raised by homocysteine treatment in microglia [41]. Furthermore, intestinal mucosal injury usually leads to the increase of cytokines including TNF-α and is related to the abnormal expression of JAK2 protein [42]. In this study, *JAK2* was a target of miR-375 and was up-regulated in UC, which is consistent with previous studies [28,29]. Its upregulation alleviated the effects of miR-375, and suppressed the proliferation and inhibited the apoptosis of YAMC cells.

However, this study has some limitations. For instance, only 30 samples of UC were obtained in this study. Therefore, we need to collect more samples to make our conclusions more accurate and reliable. Further studies are needed to focus on the regulation of SNGH5 in UC at different stages. Moreover, down-regulation of SHNG5 did not totally abolish the TNF-α-induced cell dysfunction compared with untreated cells, which may indicate that there are multiple mechanisms in UC that co-regulate to alleviate TNF-α induced dysfunction. Moreover, the effective in *vivo* experiments are needed to support the results.

## Conclusion

Our research suggested SNHG5 acted as a ceRNA to regulate the proliferation and apoptosis of YAMC cells via miR-375/*JAK2* axis. Knockdown of SNHG5 protected against TNF-α reduced dysfunction of human intestinal epithelial cells in UC. Hence, SNHG5/miR-375/*JAK2* axis can be an alternative for the treatment of UC.

## Research Highlights

- lncRNA SNHG5 was upregulated in the intestinal mucosa tissues of UC patients.
- Knockdown of lncRNA SNHG5 promoted proliferation and inhibited apoptosis of HT-29 cells.
- lncRNA SNHG5 regulated the development of UC via miR-375/*JAK2* axis.
